# Propionic acidemia: clinical course and outcome in 55 pediatric and adolescent patients

**DOI:** 10.1186/1750-1172-8-6

**Published:** 2013-01-10

**Authors:** Sarah C Grünert, Stephanie Müllerleile, Linda De Silva, Michael Barth, Melanie Walter, Kerstin Walter, Thomas Meissner, Martin Lindner, Regina Ensenauer, René Santer, Olaf A Bodamer, Matthias R Baumgartner, Michaela Brunner-Krainz, Daniela Karall, Claudia Haase, Ina Knerr, Thorsten Marquardt, Julia B Hennermann, Robert Steinfeld, Skadi Beblo, Hans-Georg Koch, Vassiliki Konstantopoulou, Sabine Scholl-Bürgi, Agnes van Teeffelen-Heithoff, Terttu Suormala, Wolfgang Sperl, Jan P Kraus, Andrea Superti-Furga, Karl Otfried Schwab, Jörn Oliver Sass

**Affiliations:** 1 Zentrum für Kinder- und Jugendmedizin, Universitätsklinikum Freiburg, Freiburg, Germany; 2 Labor für Klinische Biochemie und Stoffwechsel, Zentrum für Kinder- und Jugendmedizin, Universitätsklinikum Freiburg, Freiburg, Germany; 3 Klinische Chemie & Biochemie, Universitäts-Kinderspital Zürich, Steinwiesstrasse 75, 8032, Zürich, Switzerland; 4 Present address: University of Lausanne, Centre Hospitalier Universitaire Vaudois (CHUV), Lausanne, Switzerland; 5 Klinik für Allgemeine Pädiatrie, Neonatologie und Kinderkardiologie, Universitätsklinikum Düsseldorf, Düsseldorf, Germany; 6 Zentrum für Kinder- und Jugendmedizin, Universitätsklinikum Heidelberg, Heidelberg, Germany; 7 Klinikum der Universität München, Ludwig-Maximilians-Universität München, Munich, Germany; 8 Kinderklinik, Universitätsklinikum Hamburg-Eppendorf, Hamburg, Germany; 9 Allgemeine Pädiatrie, AKH, Medizinische Universität Wien, Wien, Austria; 10 Present address: Department of Human Genetics, Division of Clinical and Translational Genetics and Genomics; Miller School of Medicine, University of Miami, Florida, USA; 11 Stoffwechsel und Molekulare Pädiatrie, Universitäts-Kinderspital Zürich, Zürich, Switzerland; 12 Universitätsklinikum für Kinder- und Jugendheilkunde, Medizinische Universität Graz, Graz, Austria; 13 Universitätsklinik für Kinder- und Jugendheilkunde, Medizinische Universität Innsbruck, Innsbruck, Austria; 14 Klinik für Kinder- und Jugendmedizin, Universitätsklinikum Jena, Jena, Germany; 15 Present address: Helios Klinikum Erfurt, Klinik für Kinder und Jugendmedizin, Erfurt, Germany; 16 National Centre for Inherited Metabolic Disorders, Children’s University Hospital Dublin, Dublin, Ireland; 17 Allgemeine Pädiatrie, Universitätskinderklinik Münster, Münster, Germany; 18 Klinik für Pädiatrie m. S. Endokrinologie, Gastroenterologie und Stoffwechselmedizin, Charité Universitätsmedizin Berlin, Berlin, Germany; 19 Kinderklinik, Georg-August-Universität Göttingen, Göttingen, Germany; 20 Universitätsklinikum für Kinder und Jugendliche, Leipzig, Germany; 21 Klinik für Kinder- und Jugendmedizin, Braunschweig, Germany; 22 Universitäts-Kinderspital Basel, Basel, Switzerland; 23 Paracelsus Medizinische Privatuniversität, Universitätsklinikum für Kinder- und Jugendheilkunde, Salzburg, Austria; 24 Department of Pediatrics, University of Colorado, Aurora, USA

**Keywords:** Propionic acidemia, Branched-chain amino acids, Outcome, Quality of life, Clinical course, Physical development, Neurocognitive development, IQ, Long-term complications, Propionyl-coenzyme A carboxylase deficiency

## Abstract

**Background:**

Propionic acidemia is an inherited disorder caused by deficiency of propionyl-CoA carboxylase. Although it is one of the most frequent organic acidurias, information on the outcome of affected individuals is still limited.

**Study design/methods:**

Clinical and outcome data of 55 patients with propionic acidemia from 16 European metabolic centers were evaluated retrospectively. 35 patients were diagnosed by selective metabolic screening while 20 patients were identified by newborn screening. Endocrine parameters and bone age were evaluated. In addition, IQ testing was performed and the patients’ and their families’ quality of life was assessed.

**Results:**

The vast majority of patients (>85%) presented with metabolic decompensation in the neonatal period. Asymptomatic individuals were the exception. About three quarters of the study population was mentally retarded, median IQ was 55. Apart from neurologic symptoms, complications comprised hematologic abnormalities, cardiac diseases, feeding problems and impaired growth. Most patients considered their quality of life high. However, according to the parents’ point of view psychic problems were four times more common in propionic acidemia patients than in healthy controls.

**Conclusion:**

Our data show that the outcome of propionic acidemia is still unfavourable, in spite of improved clinical management. Many patients develop long-term complications affecting different organ systems. Impairment of neurocognitive development is of special concern. Nevertheless, self-assessment of quality of life of the patients and their parents yielded rather positive results.

## Background

Propionic acidemia (PA) is a rare autosomal recessively inherited inborn error of propionate metabolism. The biochemical defect involves the conversion of propionyl-coenzyme A (propionyl-CoA) to methylmalonyl-coenzyme A (methylmalonyl-CoA) by the mitochondrial enzyme propionyl-CoA carboxylase (PCC, EC 6.4.1.3). Thus, the metabolism of branched-chain amino acids, odd-numbered fatty acids, cholesterol side chains, thymine and uracil is impaired.

Most patients with this disorder present in the neonatal period with severe metabolic acidosis and hyperammonemia. However, late presentations with predominantly neurological symptoms as well as asymptomatic individuals have also been described [1-3]. With progression of the disease, patients are prone to complications affecting the neurologic, cardiologic, hematologic, immunologic and gastrointestinal system. As underlined in the proceedings of a recently held consensus conference on PA, the understanding of the disease course in PA is rather limited, with most information deriving from case reports and small retrospective case series [4]. Although few authors have reported data on larger groups of patients [2,5-9] information on the long-term outcome of affected individuals is still limited. This has prompted us to approach a comprehensive collection of outcome data on 55 PA patients from 16 metabolic centers in Germany, Austria and Switzerland. While the two preceeding reports [10,11] focussed on the question whether PA should be a target disease of neonatal screening and on investigations on gene and enzyme levels we now describe the clinical course of the disease and outcome of the patients in the study cohort. Apart from clinical information including IQ testing, assessment of growth data and endocrinological analyses, here we also address the quality of life of the patients and their families.

### Patients, materials and methods

#### Patients

We investigated 55 living patients with PA from 16 metabolic centers in Germany (12 centers), Austria (3 centers) and Switzerland (1 center). All 55 patients were examined at their respective metabolic center between September 2007 and March 2008. Due to a subproject assessing possible benefits of neonatal screening for PA, which has been described separately [10], only living patients younger than 20 years were included in this study. The 55 patients derived from 50 families and included 10 sibpairs. Median age at time of investigation was 5.2 years (range 5 days to 18.6 years). Details of the study cohort are displayed in Table 1.

**Table 1:** Sociodemographic data and details of family and perinatal history of 55 study patients


**Ethnic origin**	
Europe	51/55 (93%)
thereof Turkey	19/55 (37%)
Northern Africa	1/55 (2%)
Middle East	3/55 (5%)
**Sex**	
male	n = 35 (64%)
female	n = 20 (36%)
**Family history**	
parental consanguinity	47%
more than one child affected by PA	6/50 families (12%)
siblings known to have died from PA	4/50 families (8%)
unexplained infant death	31/50 families (62%)
**Perinatal history**	
uneventful pregnancy	37/54 (69%)
born at term	44/49 (90%)
unremarkable 10 minutes APGAR score (≥9)	44/44 (100%)
median birth weight (n = 49)	−0.01 SDS
median body length at birth (n = 48)	0.66 SDS
median head circumference at birth (n = 43)	−0.79 SDS

The study was approved by the Ethical Committee of the University of Freiburg and local ethics boards, if this was considered appropriate by the local physicians. Written informed consent was obtained from the patients and/or their parents.

#### Data collection

We collected retrospective clinical information by review of medical records. To standardize data collection a questionnaire was designed which was usually filled in by two of the authors during visits at the participating metabolic centers. The questionnaire focussed on the mode of diagnosis, number of hospital admissions, the physical and psychomotor development, long-term complications and long-term treatment regimens.

#### Clinical and laboratory investigations

Clinical examinations were performed during a routine outpatient visit at the respective metabolic clinic in the majority of patients (52/55 patients), while 3 children were investigated during a hospital stay. Within routine blood sampling, serum was obtained for the assessment of endocrine parameters. In prepubertal children those parameters comprised insulin-like growth factor 1 (IGF-1), insulin-like growth factor binding protein 3 (IGFBP3) and thyreotropin. In pubertal and postpubertal patients determinations of sexual hormones (estradiol, testosterone, follicle stimulating hormone and luteinizing hormone) were added. To evaluate growth and physical development growth data were collected retrospectively. Bone age was assessed using hand and wrist X-rays (bone age according to Greulich and Pyle [12]) and the near final height was calculated according to the tables by Bayley and Pinneau [13]. For patients of Turkish, Afghan, Lebanese, Italian, Iraqi, Algerian, Syrian and Greek origin, growth charts for Turkish children were applied.

#### Assessment of neurocognitive outcome and quality of life

In patients older than 2.5 years of age psychological testing was performed. We used two different standard tests, the Snijders-Oomen Test (SON 2 ½-7, [14]) for patients aged 2.5 to 8.5 years and the Culture-Free-Test (CFT 20-R, [15]) for patients older than 8.5 years.

To assess the quality of life of the patients and their families, 3 standardized questionnaires were applied: The “Kid-Kindl” questionnaire [16,17], evaluating the health-dependent quality of life, was filled in by or together with the patients themselves, whereas the “Strengths and Difficulties Questionnaire” [18] and the “Familien-Belastungs-Fragebogen” (= Families’ Burden Scale; [19]) were completed by the patients’ parents. The “Strengths and Difficulties Questionnaire” specifically addresses the patients’ emotional and behavioural problems while the latter one targets the families’ daily social, financial and familial burdens.

#### Statistics

All statistical analyses were performed using SPSS 16.0 and 17.0 software (IBM, Chicago, Il., USA).

## Results

This study provides data on 55 patients with PA. Details on family history and perinatal histories are given in Table 1.

### Diagnosis and clinical manifestation

Age at diagnosis ranged between 1 day and 8 years of life (median 7 days). Out of 55 patients, 35 were diagnosed by selective metabolic screening (prompted by clinical or laboratory abnormalities or by positive family history), whereas 20 patients were identified by newborn screening. Only 4 patients (7%) have remained without acute symptoms so far (age 6 months to 6.5 years). About three quarters of patients (73%) already became symptomatic within the first 5 days of life. Only 3 patients presented beyond day 90. The median age at onset was day 4 of life. Details on the clinical and laboratory findings during the initial acute decompensation have been reported previously in the context of newborn screening [10].

### Clinical course, metabolic crises, hospitalizations

The frequency of metabolic decompensations, defined as episodes of a severely reduced clinical condition or impaired consciousness which could be associated with acidosis and/or hyperammonemia, was highest within the first year of life (median 2 decompensations, range 0–8) and decreased with increasing age. 11 patients had ≥ 15 metabolic crises. 46/55 patients (84%) required intensive care treatment at least once due to metabolic decompensation and 32/55 patients (58%) required intensive care treatment more than twice. In 8 patients (15%) dialysis was performed due to hyperammonemia. Carglumic acid has not been used in any of the patients with hyperammonemic crisis.

The most common triggers of metabolic decompensation were common infections, predominantly upper respiratory tract infections or gastroenteritis. A high protein intake as an isolated trigger of metabolic crises was reported in only 3 patients.

### Physical development

Hand and wrist X-rays were available from 28 patients. In half of the patients bone age was retarded. Median difference between bone age and chronological age was −0.5 month (range −37 months to +21 months). In 8 patients (29%) bone age was accelerated, whereas in 6 patients (21%) bone age was identical with chronological age.

To assess the patients’ physical development, growth parameters from the medical records were evaluated at different ages. At birth, median height SDS was +0.66 (n = 48). However, from the age of 3 months to 10 years patients tended to be smaller (height SDS −0.21 to −1.19 at different ages) compared to population standards. At the time of examination, median height SDS was −0.49, median genetic target height (calculated from parental heights) was 0.48 SDS. The majority of patients (91%) had a height SDS within the normal range (height SDS −2.0 to 2.0) whereas only 6% of patients were small with a height SDS below −2.0. There was no significant correlation between the number of metabolic crises and growth at the age of 12 months and 3 years, respectively. Most patients did not reach their genetic target height or genetic height potential at a young age. This is reflected by a zero correlation between the SDS of the actual body height and the genetic target height as well as the SDS of the near-final height and the genetic target height of the patients. Thus, patients showed an early onset and progressive growth retardation.

The median body weight at birth was 0.01 SDS. There was a tendency toward decreased body weight within the first three months of life (median −0.92 SDS at age three months), but nearly normal values were found for the age of 1 to 10 years (median −0.64 - 0.51 SDS, range −3.86 SDS to 3.78 SDS). The body mass index (BMI) of the patients was low (−0.63 SDS to −0.17 SDS) within the first year of life, but tended to be higher in comparison with population standards from the age of 1 year (1.0 SDS-1.5 SDS). The BMI at age 3 months and 2 years did not correlate with the daily protein intake of the patients (Kendall Tau = 0.005; p = 0.875 and Kendall Tau = 0.8 * 10^-5^; p = 0.861, respectively).

The median head circumference at birth was −0.79 SDS and tended to be low throughout the first three years of life with median SDS values between −0.31 and −0.9 (range −5.1 SDS to +2.1 SDS).

### Endocrinological aspects

Median IGF-1 level (n = 44) was lower than in age- and gender-matched controls (−1.3 SDS, range −3.63 to 1.12 SDS), but did not correlate with the patients’ height. In contrast, median IGFBP3 level (n = 42) was slightly higher compared to population standards (+0.5 SDS, range −1.9 to 2.25). 4/50 patients (8%) had elevated thyreotropine levels, but there was no case of severe hypothyroidism. Sexual hormones were analyzed in 11 pubertal and postpubertal patients. They yielded normal results in 10 patients and hypergonadotropic normogonadism in one 15-year-old boy.

### Psychomotor and cognitive development

The majority of patients showed at least a mild impairment of psychomotor and cognitive development. 44% of patients who had reached kindergarten age (≥3 years, n = 45) attended a kindergarten for children with special needs. At school age (≥ 6 years), 70% of the patients (21/30) needed special education. Only 5 children attended a regular school and among them, one patient even attended a school aiming at qualification for university studies. The remaining 4 children had not yet been enrolled in school.

**Figure 1:**
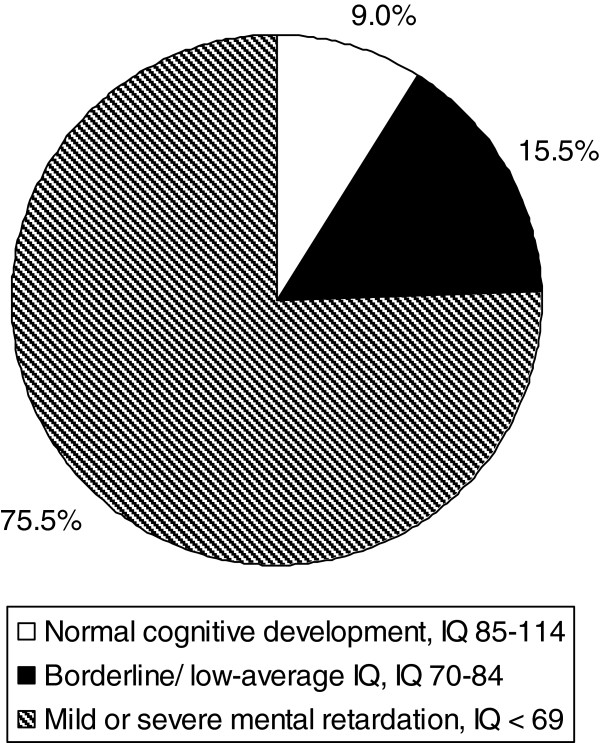
**Neurocognitive outcome in 45 patients with propionic acidemia (PA): IQ data were determined for 40 patients, 5 patients could not be tested due to severe cognitive impairment.** The latter 5 patients were classified with an IQ < 69.

An overview on the cognitive development and IQ data is given in Figure 1.

Median IQ was 55. The oldest patient with a normal IQ was 15.5 years old. Data on the cognitive development/IQ have previously been reported by Grünert et al. [10].

### Complications and outcome

Common abnormalities in our study cohort were motor retardation (55%), retardation in speech development (55%) and muscular hypotonia (51%). Less common neurologic symptoms comprised impaired hearing ability (13%), ataxia (9%) and visual impairment (7%). After completion of data collection, one patient developed bilateral optic atrophy at the age of 9.5 years. At age 11 years, she still could follow movements with her right eye and differentiate light and dark with both eyes.

Brain imaging results [magnetic resonance imaging (MRI) and computed tomography (CT)] were available of 26 patients: 11 MRI and CT scans yielded unremarkable results, whereas in 15 patients, pathologic findings were present. The most common findings (5/26, 19%) were changes in the basal ganglia [10].

As PA is a multi-system disease, complications involve many different organs and organ systems. An overview on complications identified in our study cohort is given in Table 2.

**Table 2:** Clinical complications in 51 symptomatic PA patients based on medical records

**Affected organ/organ system**	**Frequency in the study cohort of 55 PA patients**
**Bone marrow**	
Pancytopenia	11%
Neutropenia	29%
Thrombocytopenia	35%
Anemia	82%
**Central and peripheral nervous system**	
Psychomotor retardation	55%
Delayed development of the speech	55%
Muscular hypotonia	51%
Metabolic stroke	9%
Ataxia	9%
Impaired hearing ability	13%
Visual impairment	7%
**Heart**	
Long-QT syndrome	22%
Cardiomyopathy	9%
**Pancreas**	
Pancreatitis	4%
**Skeletal system**	
Osteoporosis	2%

Anemia and pancytopenia were not only found during metabolic decompensations but also while the patients were metabolically stable. The hematological abnormalities were usually reversible, but several patients had more than one episode of anemia/neutropenia. Cardiomyopathy was reported in 5 patients, including 2 with only a mild reduction of the ejection fraction. In one patient the cardiomyopathy was hypertrophic, none of the patients showed severe dilatation of the ventricle. In two patients cardiomyopathy was reversible. In one of those, a 7-year-old girl, cardiomyopathy occurred in context of a metabolic decompensation and lead to cardiorespiratory insufficiency requiring intensive care treatment.

In two patients acute pancreatitis was reported at the age of 14 and 18 months, respectively. In both children pancreatitis was associated with metabolic decompensation. In the 14-month-old patient laparotomy was performed due to concomitant ileus and suspected peritonitis. Chronic or recurrent pancreatitis was not observed in any of the study participants. Mild osteopenia diagnosed by wrist X-ray was documented in one 7-year-old patient and was treated by oral calcium supplementation.

### Long-term therapeutic regimens

#### Diet

Retrospectively obtained nutritional data were rather heterogeneous. Most patients received a diet restricted in natural protein to reduce the intake of precursors of propionyl-CoA. Within the first 5 years of life, median natural protein intake ranged from 0.8 to 1.1 g/kg body weight per day. In the majority of patients an amino acid mixture free of precursors of propionyl-CoA was supplemented to meet age-appropriate protein intake recommendations. Amino acid mixtures were administered in doses ranging from 0.1 to 4.1 g/kg body weight per day (median intake between 0.7 and 0.9 g/kg body weight per day at different ages within the first 5 years of life).

#### Feeding difficulties and percutaneous endogastric tube feeding

Feeding problems were frequent. In 27/55 (49%) patients implantation of a percutaneous endogastric tube (PEG) was necessary. PEG tube feeding was started at a median age of two years (range from 6 months to 7 years). In most patients with PEG tube the meals were predominantly given orally, whereas only 15% of patients were exclusively tube-fed (nasogastric tube or PEG tube). In 12/55 patients (22%) aversion to natural protein was observed.

#### Carnitine

To prevent secondary carnitine deficiency, L-carnitine was supplemented in most patients (93%, 51/55) with recorded doses ranging from 31 to 601 mg/kg body weight/day. In 12/36 patients (33%) a low concentration of free carnitine was documented at least once during a hospitalization (for reference values see [20]).

#### Reduction of gut bacterial flora by oral antibiotics

In 40% of patients antibiotic treatment was performed to reduce the bacterial gut population, a major source of propionic acid. In 50% of these patients metronidazole was given over a period of 2 weeks per month whereas 50% of them received metronidazole and colistin in different intervals and dosing regimens.

#### Quality of life

Patients older than 8 years (n = 20) were asked to evaluate their quality of life with regard to self-confidence/self esteem, body perception, emotional stress/emotional well-being, family life, school, friendships and their attitude towards their disease. A parameter summarizing different scales was calculated. Seven out of these 20 patients were not able to answer due to cognitive deficits. Family life was the top-ranked aspect (n = 7/13). The most distinct variations within our patient cohort were found in the category of self-confidence/self esteem and emotional stress/emotional well-being. The majority of the 13 patients appeared to consider themselves as healthy and not affected in their daily life reflected by a median KidKindl total score which did not differ significantly from the healthy control group [17].

The results of the Strengths and Difficulties questionnaire as well as corresponding data of healthy controls are displayed in Table 3. Notably, 29% (14/48) of parents have the impression that emotional problems, concentration difficulties and pathologic interaction with peers have a high impact on their child’s quality of life.

**Table 3:** Results of the strengths and difficulties questionnaire (n = 48) and frequency of abnormalities in relation to healthy controls

**SDQ subscale**	**% of abnormal findings in study cohort of PA patients**	**% of abnormal findings in healthy controls**	**Frequency of abnormal findings in PA patients in relation to healthy controls**
Emotional symptoms	25	9	2.8
Conduct problems	20	15	1.3
Hyperactivity/inattention	11	8	1.4
Peer relationship problems	41	12	3.4
Total difficulties score (generated from subscale 1–4)	28	7	4.0
	(in addition borderline abnormalities in 20%)		
Prosocial behaviour	34	3.6	9.4
Impact on child’s life	29		

The “Familien-Belastungs-Fragebogen” which especially addresses the families’ daily social, financial and familial burdens yielded highly variable results among affected families. Daily and personal burdens were considered to have the highest negative impact on family life, while coping with the disorder and the strain on siblings were not considered major problems. There was no correlation between the patient`s IQ and the strain on the families from the parents’ point of view.

## Discussion

### Clinical manifestation

As reflected in our study cohort, the clinical heterogeneity of PA is broad, ranging from neonatal onset with severe neurologic symptoms to asymptomatic children. However, most patients seem to manifest within the neonatal period. About three quarters of the study participants became symptomatic within the first 5 days of life already. Late-onset forms were rare. Asymptomatic individuals, such as the 13-year-old girl described by Wolf et al. [1], were the exception even among children identified by newborn screening who received early treatment. Similar observations have been made by other authors. Sass et al. [8] reported a prevalence of 86% early manifestations in a study of 42 PA patients whereas in a French retrospective study with 17 PA patients 71% were classified as early onset patients [21]. Of 25 PA patients reported by Lehnert et al. [6] 92% presented within the neonatal period.

### Neurologic outcome

Poor intellectual development is still the rule in PA. About three quarters of our study patients were mentally retarded. Median IQ was only 55 and the vast majority of patients required special education. This is in accordance with earlier case series [8,9]. However, our data allow a more positive view when compared to outcome studies of the 1990s [2,7]. Although improved acute and long-term management have increased the survival rates within the last decades, the neurologic outcome of PA patients is still unsatisfactory. As recently shown, the IQ is negatively correlated with the number of metabolic crises, but not simply with patients’ age [10].

Neuroradiologic findings are rather nonspecific in patients with PA ranging from white matter abnormalities to widening of sulci and fissures, delay in myelination, brain atrophy and basal ganglia changes [22-24]. In our study, brain imaging yielded pathologic findings in 42% of patients (11/26) with basal ganglia changes being the most common abnormality.

### Physical development und endocrinologic aspects

Impaired physical development is a common problem in patients with PA. Sass et al. [8] and van der Meer et al. [21] have reported a tendency towards decreased body height in PA patients with a mean height SDS of −1.19 and −2.0, respectively. In accordance with those studies, height was also decreased in our patients especially when compared to patients’ target heights calculated from parental heights. Our data are indicative of an early onset and progressive growth retardation in PA patients. In contrast, patients’ BMI were found to be higher compared to population standards after the first year of life. Failure to thrive has been postulated to be due to iatrogenic dietary protein restriction, frequent infections and metabolic decompensations [21,25]. However, in our patients growth neither correlated with patients’ daily protein intake nor with the number of metabolic decompensations. To further elucidate the cause of growth retardation, several endocrinologic parameters were determined. Secondary deficiency of IGF-1 can be caused by malnutrition or hypothyroidism [26]. IGF-1 concentrations were decreased in the majority of patients, however, significant correlation with the patients’ body height could not be shown. Touati et al. [27] also reported low IGF1 concentrations in patients with PA, but growth curves of those patients were within normal limits. Severe hypothyroidism was detected in none of our patients. Abnormal puberty which has been reported as a problem before [9] was also not found apart from one case of hypergonadotropic normogonadism.

### Complications

The most common complications in our patients were hematological abnormalities including anemia, neutropenia, thrombocytopenia or pancytopenia, findings that have been described previously [28,29]. Cardiac complications, especially cardiomyopathy, may contribute significantly to the mortality of PA patients with several fatal cases reported [30]. More recently, the association between PA and long-QT syndrome has been described [31]. Jameson and Walter [32] reported the first case of a child with PA and long-QT syndrome suffering a life-threatening event secondary to long QT syndrome. In our patients, long-QT syndrome was rather rarely reported (24% of patients) compared to data from an observational longitudinal study with 10 PA patients published by Baumgartner et al. [33] who found a prolonged QTc interval (>440 ms) in 70% of patients beyond infancy. However, ECGs were only documented for 38/55 patients of our cohort and in most cases, no exercise and 24h ECGs were performed.

Osteoporosis or osteopenia have only been documented in one patient. However, as bone density assessment is usually not done on a regular basis in most patients, osteoporosis might be underdiagnosed.

The rather high incidence of impaired hearing ability in our PA population suggests a connection between both disorders. This is in contrast to an earlier assumption by Brosch et al. [34], who reported four PA patients with sensoneurinal hearing loss.

Pancreatitis seems to be a rare complication [35,36] and was documented in two of our patients only. Optic atrophy, observed in one of the patients studied here, has been reported in four patients with PA [37,38] before.

### Therapy

There is still controversy about the optimum natural protein intake of patients with PA and different therapeutic regimens are used by different metabolic centers.

The median natural protein intake in our patients ranged from 0.8 to 1.1 g/kg body weight per day within the first 5 years, well in accordance with the recommendations given by Sass et al. [8]. Most patients’ diet was supplemented with a precursor-free amino acid mixture. L-Carnitine was supplemented in 93% of the patients. The high frequency of feeding disorders in this series of patients must be stressed and is in agreement with a report by Touati et al. [27]. About half of all patients received tube-feeding to promote growth and ensure metabolic stability.

### Quality of life

While the neurologic outcome of PA has been addressed by several case reports and small case series, very little is known about the quality of life of PA patients. In our study, there might be an ascertainment bias because the questionnaire could only be filled in by those patients whose cognitive impairment was only moderate. Among all categories, family life was the top-ranked aspect with regard to the patients’ quality of life. This may well reflect the significant role of the parents and the family ties that can arise by caring for a child that suffers from a chronic, potentially life-threatening disease. Notably, evaluation of the questionnaire data suggests, that most patients consider their quality of life high. Most patients appeared even to perceive themselves as healthy and felt not affected in their daily life. The results of this self-evaluation resemble very much the data of a healthy control group [17].

From the parents’ perspective, difficulties in the interaction of patients with peers are the most common problem. Notably, according to their parents’ estimate, abnormal findings are more common in PA patients in all categories addressed by the questionnaire when compared to healthy children. This includes emotional issues and conduct problems. One half of all patients were considered to have psychological problems or to show at least borderline abnormalities. The percentage of PA patients who – based on their parents’ view- have psychological problems was about four times higher compared to healthy controls [39].

Chronic diseases may severely affect family life and pose psychosocial stress to parents and siblings. The results of the FaBel-questionnaire revealed higher values for five of the six subscales for PA patients when compared to the healthy controls reported by Ravens-Sieberer [19]. Those scales include daily and social strains, personal strains and worries about the future, financial burdens and strains on siblings. These findings underline that apart from the medical care affected families also need psychological and social support. Therefore, they strongly advocate for the inclusion of psychologists and social workers into the interdisciplinary metabolic team.

## Conclusion

Our data on a large cohort of PA patients show that the outcome is still poor in most cases especially with regard to the neurocognitive and psychosocial development. Long-term complications affecting various organ systems are common.

## Abbreviations

BMI: Body mass index (indices); CFT: Culture-Free-Test; CoA: Coenzyme A; IGF-1: Insulin-like growth factor 1; IGFBP3: Insulin-like growth factor binding protein 3; IQ: Intelligence quotient; PA: Propionic acidemia; PCC: Propionyl-CoA carboxylase; SDS: Standard deviation score; SON: Snijders-Oomen test.

## Competing interests

This study was financially supported by Milupa Metabolics GmbH & Co KG, Friedrichsdorf, Germany, who also financed the Freiburg Workshop on Propionic Acidemia (Oct 16–18, 2008). However, the sponsor had no influence on the planning and conduction of the study and the interpretation of the results.

## Authors’ contributions

SCG participated in planning and conduction of the study and in data interpretation. She recruited patients and prepared the draft of the manuscript and the display items together with JOS. SM and LdS visited all metabolic centers involved in this study, gathered the clinical data by reviewing all available medical records, performed the IQ tests and collected the required laboratory samples. They assembled the data, performed the statistical analyses, prepared one display item, and contributed to the interpretation of the findings. MB selected and supervised the psychological/IQ tests and was the statistical advisor of the study. MW organized and performed laboratory investigations and provided logistic support. KW participated in the study design and in the evaluation of endocrinologic data. TM, ML, RE, RS, OAB, MRB, MB-K, DK, CH, IK, TM, JBH, RS, SB, HGK, VK, SS-B, AvT-H recruited patients for the study, provided clinical data, were advisors at data collection and contributed to the interpretation of the findings. TS was in charge of enzymatic diagnostic tests. WS and JPK were advisors on data evaluation, ASF advised on study design and interpretation. KOS was the main pediatric endocrinologist of the study. He supervised the endocrinologic evaluation, assessed bone ages and advised on metabolic aspects. JOS conceived and coordinated the project, drafted the manuscript together with SCG and supervised data collection and interpretation. He serves as guarantor for the article. All authors read and approved the final manuscript.
